# Trends in prevalence, health disparities, and early detection of dementia: A 10-year nationally representative serial cross-sectional and cohort study

**DOI:** 10.3389/fpubh.2022.1021010

**Published:** 2023-01-04

**Authors:** Kevin Lu, Xiaomo Xiong, Minghui Li, Jing Yuan, Ye Luo, Daniela B. Friedman

**Affiliations:** ^1^Department of Clinical Pharmacy and Outcomes Sciences, University of South Carolina, Columbia, SC, United States; ^2^Department of Clinical Pharmacy and Translational Science, University of Tennessee Health Science Center, Memphis, TN, United States; ^3^Department of Pharmacy Administration, Fudan University, Shanghai, China; ^4^Department of Sociology, Anthropology and Criminal Justice, Clemson University, Clemson, SC, United States; ^5^Department of Health Promotion, Education, and Behaviour, Arnold School of Public Health, University of South Carolina, Columbia, SC, United States

**Keywords:** mild cognitive impairment, dementia, Alzheimer's disease, early detection, middle-aged and older populations

## Abstract

**Objective:**

To identify trends in the prevalence of mild cognitive impairment (MCI) and dementia, and to determine risk factors associated with the early detection of dementia among U.S. middle-aged and older adults.

**Methods:**

We used 10-year nationally representative longitudinal data from the Health and Retirement Study (HRS) (2006–2016). Adults aged 55 years or older were included to examine the trend. To identify the associated factors, adults aged 55 years or older in 2006 who developed MCI or dementia in subsequent waves until the 2016 wave were included. Early and late detection of dementia were identified using the Langa-Weir classification of cognitive function. Multivariate logistic regression models were used to identify factors associated with the early detection of dementia.

**Results:**

The sample size for the analysis of the prevalence of MCI and dementia ranged from 14,935 to 16,115 in the six survey years, and 3,729 individuals were identified to determine associated factors of the early detection of dementia. Among them, participants aged 65 years or older accounted for 77.9%, and male participants accounted for 37.2%. The 10-year prevalence of MCI and dementia was 14.5 and 6.6%, respectively. We also found decreasing prevalence trends in MCI (from 14.9 to 13.6%) and dementia (from 7.4 to 6.0%) overall in the past decade. Using logistic regression controlling for the year, non-Hispanic black (MCI: OR = 2.83, *P* < 0.001; dementia: OR = 2.53, *P* < 0.001) and Hispanic (MCI: OR = 2.52, *P* < 0.001; dementia: OR = 2.62, *P* < 0.001) had a higher prevalence of both MCI and dementia than non-Hispanic white participants. In addition, men had a lower prevalence of MCI (OR = 0.94, *P* = 0.035) and dementia (OR = 0.84, *P* < 0.001) compared to women. Associated factors of the early detection of dementia include age, gender, race, educational attainment, stroke, arthritis diseases, heart problems, and pensions.

**Conclusion:**

This study found a decreasing trend in the prevalence of MCI and dementia in the past decade and associated racial/ethnic and gender disparities among U.S. middle-aged and older adults. Healthcare policies and strategies may be needed to address health disparities in the prevalence and take the associated factors of the early detection of dementia into account in clinical settings.

## Introduction

Dementia is a broad term used to describe a range of symptoms related to the decline in cognitive functions that impair an individual's daily and independent ability (1–3). Due to the increasing aging population, it is expected that the number of participants with dementia will triple in 2050 compared to 2010 (4). The total direct medical costs of dementia are estimated to exceed $1 trillion in the United States in 2050 (5).

Given that there is still no curative treatment for dementia, reducing risk in high-risk populations is essential to slow the surge in the prevalence of dementia and to reduce economic burdens (6, 7). Mild cognitive impairment (MCI) is a transition state between the cognitive decline of normal aging and dementia, with which individuals appear to have a significantly higher risk of dementia (8). Compared with dementia, the most important distinguishing feature of MCI is that it is reversible and treatable (9, 10). Participants with MCI progress at the rate of 5–10% yearly and about one-third may revert to normal (9). Several elements could have an impact on the reversion (10). Evidence has shown that participants with milder symptoms and without apolipoprotein E (APOE) ε4 allele are more likely to reverse to normal cognition, and healthy lifestyles including exercise and diet can prevent or reverse cognitive decline (10–12). Due to the unavailability of a specific test to confirm, MCI is generally diagnosed by doctors based on symptoms and information provided subjectively (13, 14). As a result, individuals without MCI may sometimes be misdiagnosed and treated incorrectly (10).

Detecting MCI and treating correspondingly could generate economic benefits to the healthcare system and middle-aged and older adults. A recent report from the Alzheimer's Association shows that if all individuals with Alzheimer's disease who were living in the U.S. in 2018 had been detected at the MCI stage, a total of $8 trillion would have been saved (5). However, the detection of MCI is difficult (15). Common screening tools used to detect dementia in clinical practices are not reliable in detecting MCI because the decline in the cognition of individuals with MCI can be subtle (15). Comprehensive assessments for detecting MCI based on brain imaging markers or blood-based neurochemical biomarkers are usually costly, unmodifiable, and lack the sensitivity and specificity required to detect MCI (16–18). Thus, it is critical to determine some less costly and modifiable associated factors of the detection of MCI. In addition to determining associated factors of the detection of MCI, identifying the trends in it is of great importance to implications for the risk reduction and management of dementia (16). However, there have been no reports on trends in the prevalence of MCI and its disparities. To fill the gap in the literature, we aimed to determine associated factors for the detection of MCI, and trends in its prevalence using the Health and Retirement Study (HRS) (19), a large nationally representative prospective cohort study of U.S. middle-aged and older adults.

## Methods

### Data

This study followed the STROBE guidelines for observational studies. We used 10-year longitudinal data from waves 8 (2006) to 13 (2016) from the original HRS dataset and the RAND HRS v1 longitudinal file 2018. HRS is an ongoing longitudinal population-based survey sponsored by the National Institute on Aging (NIA) and Social Security Administration (SSA), which includes the representative data for adults aged 55 years old in the U.S. for our study period (19). The RAND HRS is a subset of the HRS published by the RAND Corporation with cleaned versions of variables of the HRS (19, 20). The sample of HRS is built over time. Initially, HRS recruited an initial cohort in 1992 with participants born in 1931–1941 (21). Two years later, another cohort born before 1924 was recruited. In 1998, two additional birth cohorts, born in 1924–1930 and 1942–1947, respectively, were recruited to fill the age gap and to create a representative sample of the US population over 50 (21). After that, HRS added a new cohort every 6 years to maintain the sample's representativeness (21). In addition, in 2010, HRS expanded the minority sample, which is referred to as minority oversample (21). The data and materials for HRS can be accessed at its website (http://hrsonline.isr.umich.edu/). Multistage probability sampling design with clustering was used in the HRS to identify household units as the primary sampling unit. Interviews of the HRS respondents, and their spouses if available, were conducted every 2 years (22). The HRS collects information on demographic and socioeconomic factors, health status, and cognition (19, 22). Telephone Interview for Cognitive Status (TICS) in the HRS is specifically used to identify the cognition status of participants (23). In addition, to ensure representativeness at the population level, HRS provides sampling weights for differential selection probabilities for racial/ethnic and birth cohorts and to correct for differential non-response (24). The sampling weights that the HRS provides can also be used to calculate national estimates (24).

### Study sample

To have nationally representative samples (25, 26), adults aged 55 years or older in wave 8 (2006) and the refreshment samples of waves 10 (2010) and 13 (2016) were included to examine trends in the prevalence of MCI and dementia. For identifying associated factors of the early detection of dementia, adults aged 55 years or older in wave 8 (2006) were used as the baseline, while the subsequent waves through 2016 were referred to as the follow-up. Participants were included if they had no MCI or dementia at baseline and developed MCI or dementia during the follow-up. Those with missing information on associated factors and non-positive weight information at the baseline were excluded.

### Measurement

The early and late detection of dementia were measured using the Langa-Weir Classification of Cognitive Function (LWCCF) based on TICS scores (20, 23). The total TICS score ranges from 0 to 27 points, which comprises an immediate and a delayed 10-noun free recall test (0–20 points), a serial sevens subtraction test (0–5 points), and a counting backward test (0–2 points) (20). The LWCCF is a performance measurement to determine if participants have cognitive impairments, which is derived from the Aging, Demographics and Memory Study (ADAMS), a sub-study of HRS (20). The LWCCF categorized participants who scored 0–6 points as “demented,” 7–11 as “cognitively impaired but not demented (CIND),” and 12–27 as “normal.” Given that in the ADAMS, the CIND is defined the same as MCI (20), participants with normal cognition who developed only MCI without dementia at any wave of the follow-up were classified as having the early detection of dementia, while those who had developed dementia at any wave of the follow-up were classified as having the late detection of dementia.

Potential associated factors measured in this study included demographic and socioeconomic factors, physical health factors, and health behaviors. Demographic factors included: age, gender, race/ethnicity, census regions, and educational attainment. Socioeconomic factors included total household income, public health insurance status, private health insurance status, and pension status. Physical health factors included self-reported health status, body mass index (BMI), and comorbidities (hypertension, cancer, lung diseases, arthritis diseases, diabetes, heart problems, and stroke). Health behaviors included physical activities (vigorous, moderate, and light physical activities), preventative health service use, alcohol assumption, and currently smoking. All of the potential associated factors were identified based on the HRS surveys. Specifically, physical activities were classified on a survey question about the frequency of activities of participants. Participants were identified as having vigorous, moderate, and light physical activities if they had at least 1–3 times of each activity every month. Preventative health service was based on yearly preventive health care services, including flu shots, breast cancer, mammogram, pap smear, and prostate cancer tests. Alcohol users were identified if they drank at least 1 day each week, and smokers were identified if they were currently smoking.

### Statistical analyses

The prevalence of MCI was measured as the number of participants with MCI divided by the number of participants who finished TICS in each wave. Logistic regression models controlling for the effect of survey year were used to examine gender and/or racial/ethnic disparities in the prevalence of MCI and dementia. We used two-tailed *t*-tests for continuous variables and two-tailed Chi-square tests for categorical variables to compare the baseline characteristics between the participants having an early detection of dementia and those having a late detection of dementia. We estimated a multivariate logistic regression model to identify associated factors for the early detection of dementia. All tests were based on an α of 0.05 to assess statistical significance. Data cleaning and analysis were conducted between May 2020 and June 2021, and performed using SAS Software version 9.4 (Statistical Analysis Systems, Cary, NC). Survey sampling weights were used to generate national estimates for both prevalence and regression. Specifically, variables per weight, strata, and psu in the RAND HRS dataset and coding statements “proc surveyfreq” and “proc surveylogistic” in SAS were used to conduct the weighted analysis. Since the HRS is publicly available and all participants were de-identified, this study was reviewed and exempted by the University of South Carolina Institutional Review Board (IRB).

## Results

**Figure 1:**
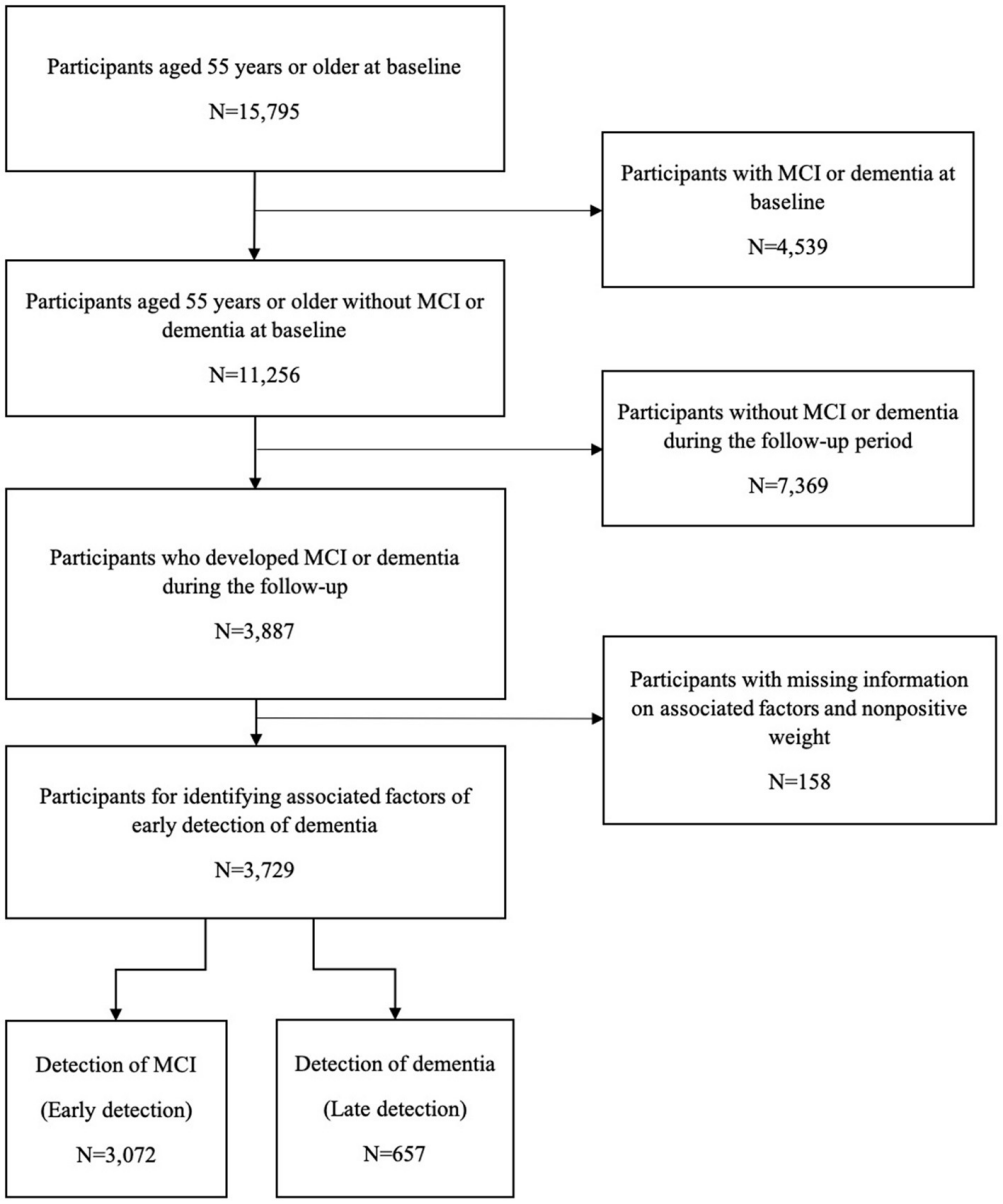
Flowchart of study sample selection. MCI, mild cognitive impairment.

To estimate the national prevalence of MCI in each wave, 15,178 participants in the 8th wave, 14,935 in the 9th wave, 16,115 in the 10th wave, 16,245 in the 11th wave, 15,941 in the 12th wave, and 15,929 in the 13th wave were, respectively, included. For identifying associated factors of the early detection of dementia, 3,729 individuals who had developed MCI or dementia during the follow-up were identified out of 15,795 participants aged 55 years or older with normal cognition at the baseline (Figure 1). Participants aged 65 years or older accounted for 77.9%, and the male participants accounted for 37.2%. Among the participants included, a total of 3,072 (82.4%) were classified as having an early detection of dementia and 657 (17.6%) as having a late detection of dementia.

**Figure 2:**
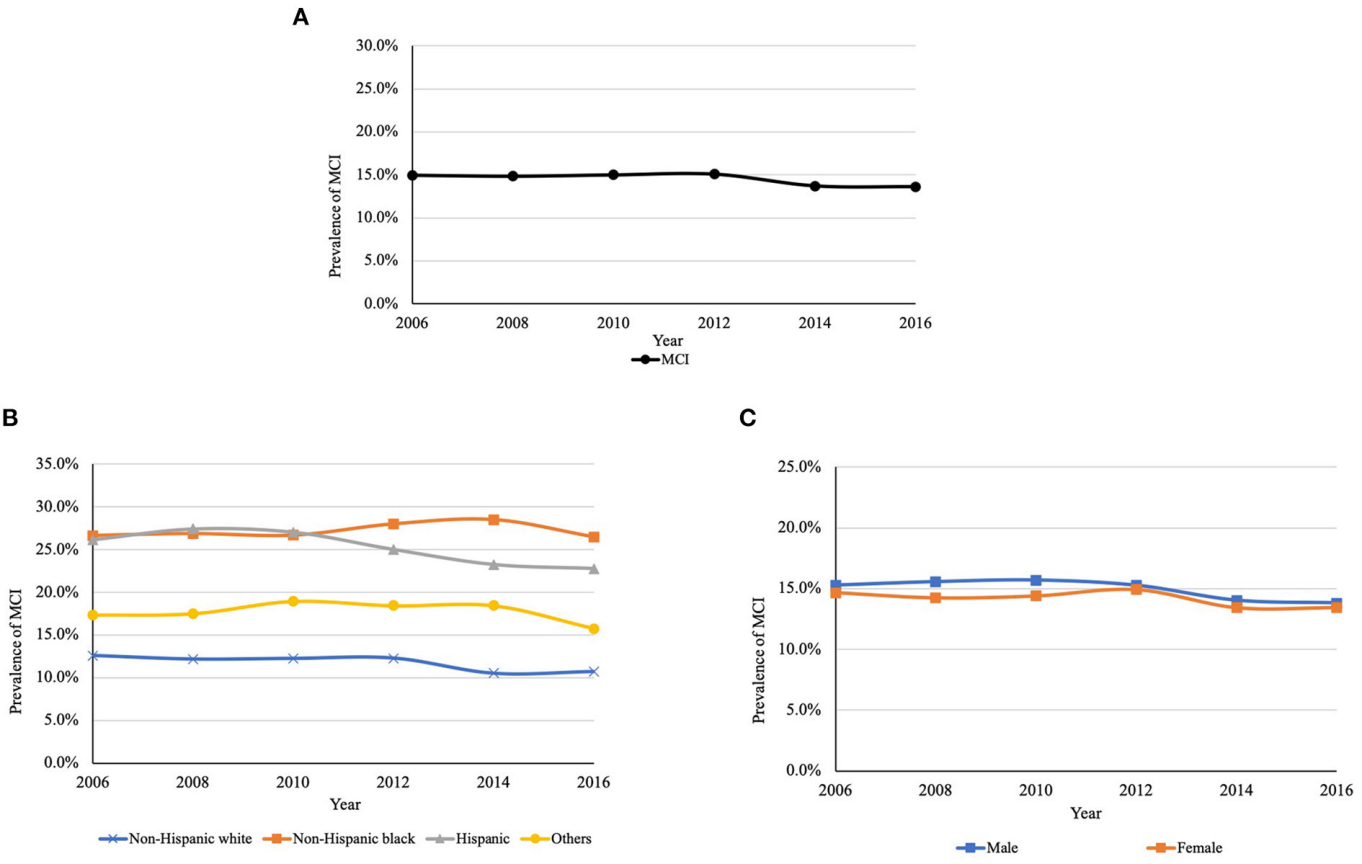
**(A)** Trends in the prevalence of MCI between 2006 and 2016. **(B)** Trends in the prevalence of MCI between 2006 and 2016 by race/ethnicity^a^. **(C)** Trends in the prevalence of MCI between 2006 and 2016 by gender^b^. MCI, mild cognitive impairment. ^a^*P* < 0.001; ^b^*P* = 0.448. *P*-values are for the type 3 test for the difference in the prevalence among race and gender.

**Figure 3:**
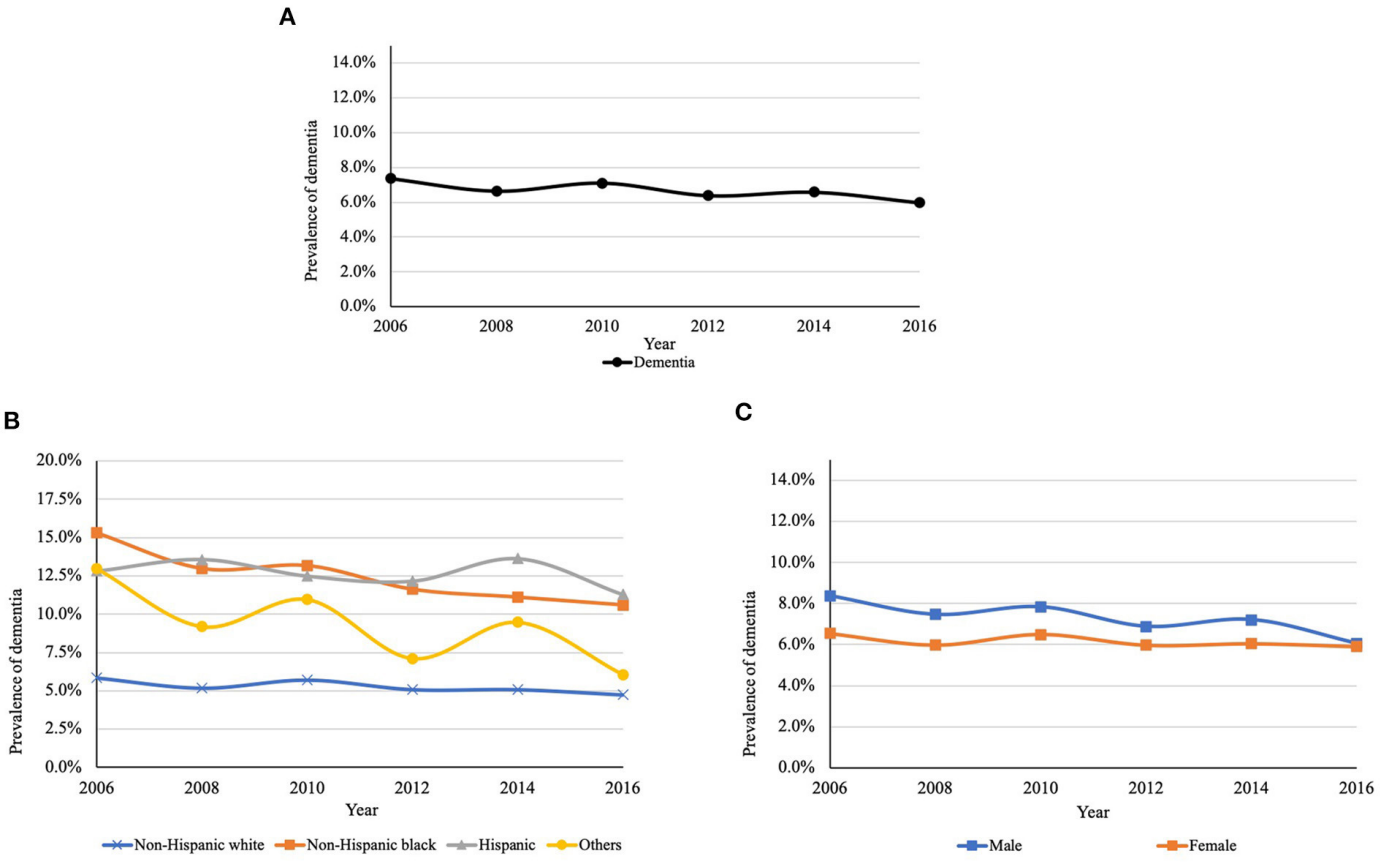
**(A)** Trends in the prevalence of dementia between 2006 and 2016. **(B)** Trends in the prevalence of dementia between 2006 and 2016 by race/ethnicity^a^. **(C)** Trends in the prevalence of dementia between 2006 and 2016 by gender^b^. ^a^*P* < 0.001; ^b^*P* < 0.001. *P*-values are for the type 3 test for the difference in the prevalence among race and gender.

Figures 2A, 3A present the overall estimated trend in the prevalence of MCI and dementia from 2006 to 2016. The 10-year prevalence of MCI and dementia was 14.5% and 6.6%, respectively. The prevalence of MCI ranged from 13.6 to 15.1%, and it was lower in 2016 (13.6%) than in 2006 (14.9%). The prevalence of dementia was decreasing over the decade, specifically, from 7.4% in 2006 to 6.0% in 2016. Figures 2B, 3B demonstrate trends in the prevalence of MCI and dementia by race/ethnicity. Using logistic regression after controlling for the year, non-Hispanic black (MCI: OR = 2.83, *P* < 0.001; dementia: OR = 2.53, *P* < 0.001), Hispanic (MCI: OR = 2.52, *P* < 0.001; dementia: OR = 2.62, *P* < 0.001), and other racial participants (MCI: OR = 1.63, *P* = 0.244; dementia: OR = 1.77, *P* = 0.677) had a higher prevalence of MCI and dementia than non-Hispanic white participants. Figures 2C, 3C show trends in the prevalence of MCI and dementia by gender. Although there was no difference in MCI, men had a lower prevalence of MCI (OR = 0.94, *P* = 0.035) and dementia compared to women (OR = 0.84, *P* < 0.001).

The baseline characteristics of participants with detection of MCI or dementia during the follow-up are shown in Table 1. Compared to participants with late detection of dementia, those with early detection were more likely to be younger (*p* < 0.001), male (*p* < 0.001), Non-Hispanic white (*p* = 0.020), married/partnered (*p* = 0.015), with higher education (*p* < 0.001), with higher income (*p* < 0.001), without strokes (*p* = 0.002), alcohol consumers (*p* = 0.002), without public insurance (*p* < 0.001), and with private insurance (*p* = 0.027).

**Table 1:** Descriptive characteristics of baseline study sample between late and early detection of dementia.

	**Late detection**	**Weighted %**	**Early detection**	**Weighted %**	***P*-value**
	***N* = 2,394,022, Weighted no**.		***N* = 12,200,793, Weighted no**.		
**Age**					<0.001
55–64	529,792	22.1	4,329,813	35.5	
65–74	824,781	34.5	4,110,055	33.7	
75–84	797,827	33.3	2,976,825	24.4	
85+	241,622	10.1	784,100	6.4	
**Gender**					<0.001
Male	890,334	37.2	5,397,865	44.2	
Female	1,503,688	62.8	6,802,928	55.8	
**Race/ethnicity**					0.020
Non-Hispanic white	1,774,924	74.1	9,704,645	79.5	
Non-Hispanic black	297,679	12.4	1,283,193	10.5	
Hispanic	254,553	10.6	954,673	7.8	
Others	66,866	2.8	258,282	2.1	
**Census region**					0.321
Northwest	425,763	17.8	1,989,030	16.3	
Midwest	585,756	24.5	3,156,579	25.9	
South	920,887	38.5	4,458,133	36.5	
West	461,616	19.3	2,597,051	21.3	
**Marital status**					0.015
Married/partnered	1,338,800	55.9	7,591,594	62.2	
Single/separated/widowed	1,055,222	44.1	4,609,199	37.8	
**Educational attainment**					<0.0001
Lower than high school graduate	883,709	36.9	3,123,428	25.6	
High school graduate	766,616	32.0	4,329,403	35.5	
Higher than high school graduate	743,697	31.1	4,747,962	38.9	
**House income**					<0.001
$0–$19,999	890,779	37.2	3,234,627	26.5	
$20,000–$49,999	940,978	39.3	5,039,221	41.3	
≥ $50,000	562,265	23.5	3,926,945	32.2	
**Health status**					0.065
Fair/poor	819,950	34.2	3,537,121	29.0	
Good/very good/excellent	1,574,072	65.8	8,663,672	71.0	
**BMI**					0.077
< 18.5	49,764	2.1	271,950	2.2	
18.5–24.9	838,082	35.0	3,549,450	29.1	
25.0–29.9	795,379	33.2	4,515,213	37.0	
≥30.0	710,797	29.7	3,864,180	31.7	
**Chronic conditions**					
Hypertension	1,292,746	54.0	6,863,612	56.3	0.338
Cancer	297,682	12.4	1,566,740	12.8	0.790
Lung diseases	204,117	8.5	1,144,536	9.4	0.564
Arthritis diseases	1,395,642	58.3	7,570,178	62.0	0.157
Diabetes	540,735	22.6	2,384,353	19.5	0.196
Heart problems	509,910	21.3	2,976,944	24.4	0.162
Stroke	244,716	10.2	814,633	6.7	0.002
**Physical activities**
Light physical activities	2,105,911	88.0	11,117,081	91.1	0.246
Moderate physical activities	1,832,625	76.6	9,665,120	79.2	0.291
Vigorous physical activities	784,299	32.8	4,311,347	35.3	0.084
**Preventative health behaviors**					0.141
No	55,772	2.3	492,880	4.0	
Yes	2,338,250	97.7	11,707,913	96.0	
**Alcohol consumption**					0.002
No	1,777,768	74.3	8,278,532	67.9	
Yes	616,254	25.7	3,922,261	32.1	
**Currently smoking**					0.239
No	2,100,934	87.8	10,430,064	85.5	
Yes	293,088	12.2	1,770,729	14.5	
**Public health insurance**					<0.001
No	395,482	16.5	3,582,956	29.4	
Yes	1,998,540	83.5	8,617,837	70.6	
**Private health insurance**					0.027
No	1,081,512	45.2	4,801,226	39.4	
Yes	1,312,510	54.8	7,399,567	60.6	
**Pension**					0.074
No	1,550,868	64.8	8,395,012	68.8	
Yes	843,154	35.2	3,805,781	31.2	

BMI, body mass index.

Associated factors for early vs. late detection of dementia are shown in Table 2. Compared with individuals aged 55–64 years, those aged between 75 and 84 years [Odds ratio (OR) = 0.55, 95% CI Confidence interval (CI), 0.31–0.98], and 85 and older (OR = 0.49, 95% CI, 0.26–0.92) were less likely to be associated with the early detection of dementia rather than the late detection. Women were less likely to have an early detection compared to men (OR = 0.79, 95% CI, 0.65–0.96). Compared to non-Hispanic white participants, non-Hispanic participants were less likely to have an early detection of dementia other than a late detection (OR = 0.71, 95% CI, 0.54–0.94). Compared to participants with less than a high school education, those with a high school education (OR = 1.58, 95% CI, 1.22–2.05) or higher (OR = 1.74, 95% CI, 1.35–2.24) were more likely to have an early detection of dementia other than a late detection. In addition, individuals who had arthritis diseases (OR = 1.34, 95% CI, 1.06–1.70), heart problems (OR = 1.37, 95% CI, 1.05–1.78) were more likely, while those with stroke (OR = 0.69, 95% CI, 0.48–0.99) were less likely to have an early detection of dementia. Also, compared to individuals without pensions, those who receive pensions were less likely to have an early detection of dementia (OR = 0.80, 95% CI, 0.65–0.98).

**Table 2:** Associated factors of early detection compared to late detection of dementia.

	**OR (LL, UL)**
**Age**	
55–64	Ref.
65–74	0.78 (0.46, 1.33)
75–84	0.55 (0.31, 0.98)^*^
85+	0.49 (0.26, 0.92)^*^
**Gender**	
Male	Ref.
Female	0.79 (0.65, 0.96)^*^
**Race/ethnicity**	
Non-Hispanic white	Ref.
Non-Hispanic black	0.71 (0.54, 0.94)^*^
Hispanic	0.77 (0.54, 1.11)
Others	0.61 (0.29, 1.27)
**Census region**	
Northwest	Ref.
Midwest	1.10 (0.88, 1.38)
South	1.04 (0.83, 1.29)
West	1.17 (0.90, 1.54)
**Marital status**	
Married/partnered	Ref.
Single/separated/widowed	1.15 (0.90, 1.46)
**Educational attainment**	
Lower than high school graduate	Ref.
High school graduate	1.58 (1.22, 2.05)^**^
Higher than high school graduate	1.74 (1.35, 2.24)^**^
**House income**	
$0–$19,999	Ref.
$20,000–$49,999	1.33 (0.92, 1.93)
≥ $50,000	1.37 (0.96, 1.95)
**Health status**	
Fair/poor	Ref.
Good/very good/excellent	1.20 (0.90, 1.60)
**BMI**	
< 18.5	1.26 (0.65, 2.45)
18.5–24.9	Ref.
25.0–29.9	1.29 (0.95, 1.74)
≥30.0	1.23 (0.93, 1.64)
**Chronic conditions**	
Hypertension	1.19 (0.98, 1.45)
Cancer	1.09 (0.82, 1.44)
Lung diseases	1.14 (0.80, 1.62)
Arthritis diseases	1.34 (1.06, 1.70)^*^
Diabetes	0.85 (0.63, 1.15)
Heart problems	1.37 (1.05, 1.78)^*^
Stroke	0.69 (0.48, 0.99)^*^
**Vigorous physical activities**	
No	Ref.
Yes	0.89 (0.72, 1.10)
**Moderate physical activities**	
No	Ref.
Yes	0.97 (0.72, 1.32)
**Light physical activities**	
No	Ref.
Yes	1.30 (0.86, 1.99)
**Preventative health behaviors**	
No	Ref.
Yes	0.70 (0.31, 1.57)
**Alcohol consumption**	
No	Ref.
Yes	1.18 (0.96, 1.45)
**Currently smoking**	
No	Ref.
Yes	1.12 (0.79, 1.58)
**Public health insurance**	
No	Ref.
Yes	0.68 (0.42, 1.12)
**Private health insurance**	
No	Ref.
Yes	0.99 (0.81, 1.21)
**Pension**	
No	Ref.
Yes	0.80 (0.65, 0.98)^*^

BMI, body mass index; LL, lower limit; UL, upper limit.

* *p* < 0.05,

** *p* < 0.001.

## Discussion

We examined a cluster of associated factors of the early detection of dementia and reported updated trends in the prevalence of MCI and dementia using a nationally representative longitudinal dataset from 2006 to 2016. The prevalence of MCI and dementia declined from 2006 to 2016 in the U.S. which is similar to previous years or other regions (27–32). According to a review of global trends in the prevalence and incidence of dementia, although studies on trends in the prevalence are not clear, the incidence of dementia may be declining in high-income countries (27). The decline in prevalence in the U.S. between 2006 and 2016 might be the long-term result of the decline in the incidence (33). Similar to our results, a study using HRS based on previous years in the U.S. found that the prevalence of dementia declined from 1993 to 2002, and declined in 2021 compared with 2000 in the U.S. (28, 32).

Our results are also consistent with other studies conducted during a similar period of study time (29–31). Using the 2011–2015 National Health and Aging Trends Study, Freedman et al. found that there was a decline in the prevalence of probable dementia over this period, from 10.6% in 2011 to 9.9% in 2015 (29). Similarly, a study of the population in aged care services in Australia found that the age- and sex-standardized prevalence of dementia declined from 2005 to 2014 (30). In addition, Taudorf et al., using national registry-based data which included over 2 million people in Demark, showed a reduction in the incidence of dementia from 2004 to 2015 (31).

We also found that non-Hispanic black and Hispanic participants have a higher prevalence of MCI and dementia than non-Hispanic white participants. These results indicate that potential health inequities might exist among different racial and ethnic groups (34–37). Similarly, a recent study using three different algorithms for identifying dementia found that black and Hispanic participants in HRS had a higher prevalence of dementia compared to black participants (38). Policies and strategies for cognitive impairment care should be developed to ensure high-risk groups have access to healthcare resources for MCI and dementia and to reduce potential inequities (39, 40). In addition, we found that there were gender disparities in the prevalence of MCI and dementia. These findings are consistent with previous studies in different groups of individuals. Sachdev et al. found that there is no significant gender difference in the prevalence of MCI among adults aged 60 years or older, and a systematic review found that the prevalence was greater in women than in men (41, 42). This might be because MCI occurs early, while dementia comes later in age, and because women have a longer life span than men, they thus have a higher prevalence of dementia (43–45). A study by Petersen et al. also found that among adults aged between 70 and 89 years, the prevalence of MCI was higher in males than in females among older adults (46).

In terms of associated factors, variables significantly associated with the early detection of dementia compared with the late detection included younger age, male gender, non-Hispanic whites, higher educational attainment, no history of stroke, and not yet receiving pensions. The prevalence and the risk of dementia increase with aging, leading to a decrease in the probability of detecting dementia at an early stage (47, 48). Therefore, screening in the younger population for cognitive impairment is critical to avoid detecting dementia at a late stage when it cannot be reversed. Consistent with the disparities found in the trends of MCI and dementia, there were racial/ethnic and gender disparities in detecting dementia at different stages, which might be related to possible physiologically-based differences in the development of dementia (49). We also found that adults with higher educational attainment were more likely to have the early detection of dementia instead of the late detection. Tailored educational sessions might be needed for the less educated middle-aged and older adults to facilitate the early detection of cognitive impairment. The results of our study also demonstrated that several chronic diseases, including arthritis diseases and heart problems, were associated factors of the early detection of dementia other than the late detection of dementia among U.S. middle-aged and older adults. Compared with those without these chronic conditions, adults with these chronic conditions are more likely to use more healthcare services and therefore making it more likely to detect dementia at an early stage (50, 51). Therefore, for middle-aged and older adults who do not frequently use health care services, policies or measures might be tailored to encourage them to undergo regular screening or annual wellness visits for early detection of cognitive impairment. Given that routine screening for older adults has not yet been fully implemented in the U.S., our results may support the significance of this initiative. In addition to arthritis and heart problems, we found that stroke was associated with a lower possibility of the early detection of dementia, which might be related to reduced consciousness and thinking ability among participants with stroke (52). Therefore, clinical care for participants with stroke should be intensified to prevent them from the onset of dementia or reduce their potential loss caused by the onset of dementia. We also found that middle-aged and older adults with pensions were less likely to have the early detection of dementia. This is consistent with previous research showing that paid work as a productive activity reduces the risk of cognitive decline although it is also possible that this relationship is due to reverse causation that one may retire later due to stellar cognitive health or earlier because of the occurrence of MCI or dementia (53).

Our study has several important strengths. First, by using a nationally representative survey, we estimated the national prevalence of MCI in 10 years and found racial/ethnic disparities. Second, we used a longitudinal design to identify associated factors of the early detection compared with late detection of dementia, which could imply a possible causal relationship between associated factors and the early detection.

However, there are still several limitations in this study. First, although we excluded participants with MCI or participants with dementia at the baseline, we could not exclude participants who had MCI before 2006 but converted back to normal cognition in 2006, which might cause a latency bias to a certain degree. In addition, because participants with missing information on predictors and weight were excluded in the data cleaning process, there might be non-differential classification bias, which might lead the effects being underestimated. However, since only about 100 observations were excluded, it would not substantially affect the results. Second, we only included information about the living region. Due to the lack of related data, we did not include information on whether participants lived in rural or urban areas, which might be a potentially associated factor for the detection of MCI (25). Third, as we used survey data as the data source, which did not contain specific diagnostic information, we classified cognitive status based on the LWCCF and derived the total cognitive scores from telephone interviews instead of clinical diagnosis. The mapping methods that LWCCF used to categorize cognitive status and telephone interviews might lead to some biases, such as differential misclassification and response bias (20). However, as the Langa-Weir classification has been validated with HRS participants and our results are consistent with previously published studies using other data sources, such as registries (31, 54), we believe that the Langa-Weir classification was valid in determining dementia and MCI in our study. Yet, studies using specific diagnostic information are still needed to provide a more rigorous national estimate of the prevalence of dementia in the United States. Furthermore, the influence of other life events (lack of sleep, medications, drugs, etc.) on cognitive performance at the time of TIC administration could not be assessed because of a lack of data. Finally, since we used a survey-based dataset that did not include the specific diseases of each participant, we were not able to use a comprehensive comorbidity indicator to control for comorbidities. However, we have included the main comorbidities based on previously published literature, which could minimize the impact of comorbidities on other associated factors (54–57).

## Conclusion

Using a nationally representative sample of U.S. middle-aged and older adults, we found racial/ethnic disparities in the prevalence of MCI and dementia, gender disparities in the prevalence of dementia, and associated factors of an early detection compared with a late detection of dementia. Healthcare policies and strategies may be needed to address health disparities in the prevalence of MCI and dementia, as well as to take the associated factors of early detection of dementia into account in the clinical settings to reduce possible disease and economic burden due to late detection of dementia.

## Data availability statement

Publicly available datasets were analyzed in this study. The data can be found and accessed at: https://hrs.isr.umich.edu/about.

## Ethics statement

The studies involving human participants were reviewed and approved by University of South Carolina Institutional Review Board (IRB). Written informed consent for participation was not required for this study in accordance with the national legislation and the institutional requirements.

## Author contributions

KL had full access to all of the data in the study, takes responsibility for the integrity of the data, the accuracy of the data analysis, and contributed to concept and design. KL, XX, ML, JY, YL, and DF contributed to acquisition, analysis, or interpretation of data, drafting of the manuscript, and critical revision of the manuscript for important intellectual content. KL and XX contributed to statistical analysis. All authors contributed to the article and approved the submitted version.
